# *De Novo* CSNK2B Mutations in Five Cases of Poirier–Bienvenu Neurodevelopmental Syndrome

**DOI:** 10.3389/fneur.2022.811092

**Published:** 2022-03-16

**Authors:** Qi Yang, Qinle Zhang, Shang Yi, Zailong Qin, Fei Shen, Shang Ou, Jingsi Luo, Sheng He

**Affiliations:** ^1^Guangxi Key Laboratory of Birth Defects Research and Prevention, Guangxi Key Laboratory of Reproductive Health and Birth Defects Prevention, Maternal and Child Health Hospital of Guangxi Zhuang Autonomous Region, Nanning, China; ^2^Department of Genetic and Metabolic Central Laboratory, Maternal and Child Health Hospital of Guangxi Zhuang Autonomous Region, Nanning, China

**Keywords:** CSNK2B, seizure, intellectual disability, short stature, growth hormone (GH) therapy

## Abstract

The Poirier–Bienvenu neurodevelopmental syndrome is an autosomal dominant disorder characterized by intellectual disability and epilepsy. The disease is caused by mutations in the *CSNK2B* gene, which encodes the beta subunit of casein kinase II, and it has important roles in neuron development and synaptic transmission. In this study, five Chinese patients were diagnosed with Poirier–Bienvenu neurodevelopmental syndrome caused by *CSNK2B* mutations by whole exome sequencing. We detected four different *de novo* variants of the *CSNK2B* gene in these five unrelated Chinese patients: two novel mutations, namely, c.100delT (p.Phe34fs^*^16) and c.158_159insA (p.Asp55fs^*^4), and two recurrent mutations, namely, c.1A>G (p.Met1?) and c.332 G >C (p.R111P). All five patients showed mild-to-profound intellectual disabilities/or learning disabilities and developmental delays, with or without seizures. Although intellectual disability/developmental delay and epilepsy are the most common manifestations of CSNK2B deficiency, the clinical phenotypes of probands are highly variable, and there is no significant correlation between genotype and phenotype. An abnormal stature may be another common manifestation of CSNK2B deficiency. Here, we report the effects of growth hormone (GH) therapy on the patients' linear height. In conclusion, Poirier–Bienvenu neurodevelopmental syndrome is a highly heterogeneous disease caused by mutations in the *CSNK2B* gene. The phenotype was highly variable, and no significant correlation of genotype and phenotype was found. Patients with short-stature and CSNK2B deficiency may benefit from GH therapy. The identification and characterization of these novel variants will expand the genotypic and phenotypic spectrum of Poirier–Bienvenu neurodevelopmental syndrome.

## Introduction

Poirier–Bienvenu neurodevelopmental syndrome (MIM 618732) is a very rare autosomal dominant disorder characterized by early-onset seizures and variably impaired intellectual development, and it is caused by the deficiency of the casein kinase 2β (1, 2), *CSNK2B* (*CSNK2B*; MIM 115441, NM_001320.6), which is ~4 kb in length, contains seven coding exons, and is located on chromosome 6p21.33. It encodes the β subunit of the casein kinase II (CK2) protein complex, and participates in biological processes including signal transduction, metabolic processes, replication, transcription, and translation (3–8). *CSNK2B* is abundantly expressed in the brain, especially in neurons and neuroepithelial cells (9), and it has essential roles in the development of neuronal processes (10, 11). *CSNK2B* deficiency alters neuron development and synaptic transmission, resulting in severe neurodevelopmental deficiencies (12, 13). Recently, 57 unrelated patients with Poirier–Bienvenu neurodevelopmental syndrome and *de novo* mutations in the *CSNK2B* gene have been described (1, 2, 14–20). The phenotypes of these patients were heterogeneous and included treatable or untreatable seizures, mild-to-profound intellectual disabilities/or learning disabilities, language delays, and other symptoms (1, 2, 14–20). The severity of the phenotypes caused by mutations in the *CSNK2B* gene and the treatment of patients with Poirier–Bienvenu neurodevelopmental syndrome remain to be fully explored.

Here, we present five unrelated Chinese patients diagnosed with Poirier–Bienvenu neurodevelopmental syndrome, and the phenotypes were highly variable. Molecular analyses identified four *CSNK2B* variants, namely, two novel variants and two recurrent variants. We also evaluated the efficacy of growth hormone (GH) therapy and summarized the genotypes, phenotypes, and clinical features of Poirier–Bienvenu neurodevelopmental syndrome.

## Materials and Methods

### Editorial Policies and Ethical Considerations

Peripheral blood was collected from 1,230 individuals with early childhood-onset epilepsy and/or intellectual disability and the patients' family medical histories were investigated. Written informed consent for the publication of data was obtained from the patients' family. This study was approved by the Department of Genetic Metabolic Central Laboratory of Maternal and Child Health Hospital of Guangxi Zhuang Autonomous Region.

### Whole Exome Sequencing and Sanger Sequencing

Genomic DNA was extracted from 5 ml of peripheral blood samples using the Lab-Aid DNA kit (Zeesan Biotech Co., Ltd., Xiamen, China). Whole exome sequencing was performed using the Agilent SureSelect Human Exon V6 kit (Agilent Technologies, Santa Clara, CA, USA), according to the manufacturer's protocol. The prepared libraries were sequenced by the HiSeq 2500 system (Illumina, San Diego, CA, USA), with a read depth of 120 × and a sequencing depth > 20 × for 95% of the captured regions. The sequenced reads were mapped to the human reference genome assembly build hg19 GRCh37 with the Burrows–Wheeler Aligner (http://bio-bwa.sourceforge.net/). Data analysis was performed with TGex software (LifeMap Sciences, Alameda, VA, USA). Variants with minor allele frequencies of <5% in the gnomAD database (http://gnomad.broadinstitute.org/) were selected. Synonymous variants and intronic variants not located within the splice site regions were removed, and all the protein-altering variants [non-synonymous single nucleotide polymorphisms (SNPs) and indels] were systematically evaluated. The effects of the mutations were examined using PolyPhen-2 (http://genetics.bwh.harvard.edu/pph2/), SIFT (http://provean.jcvi.org/), and CADD (https://cadd.gs.washington.edu/snv), (https://cadd.gs.washington.edu/snv) databases. The pathogenicity of each variant in the patient was scored according to American College of Medical Genetics and Genomics/Association for Molecular Pathology (ACMG/AMP) guidelines (21).

## Results

### Clinical Features

Each Chinese patient was the first child of healthy parents without significant medical family history and diagnosed by whole exome sequencing. All patients underwent uneventful full-term gestations. Patients with a mutation in the *CSNK2B* gene are summarized in Table 1.

**Table 1:** Genotype and phenotype details for individuals with *CSNK2B* variants.

	**Sex Age**	**Variant**	**Height**	**ID/LD**	**Develpment**	**Age at seizure**	**Seizure types**	**EEG**	**MRI**	**ASM trialed**	**Reference**
**1**	Male 10 y	c.367+2T>C		Mild ID	Speaks only several words; w = 2 y	None	N.A.	N.A.	Normal	N.A.	(1)
**2**	Male 19 y	c.175+2T>G		Mild ID	Delayed speech; w = 18 m	18 m	^*^Myoclonic	Generalized spike/(poly) spike waves discharges and a slow background	Normal	LTG,VPA then **LEV**, **CLB**, **ZNS**	
**3**	Female 6 y 2 m	c.560 T >G (p.Leu187Arg)	<5th centile	Profound ID	Sitting without support at 1.5 y; still cannot walk	1 y	^*^Myoclonus, only twice of GTC	Diffuse spike-slow, multiple spike-slow, and slow wave discharging; frequent myoclonic seizures	Normal	VPA, clonazepam, then LEV with TPM	(2)
**4**	Female 3 y 5 m	c.620_621insC (p.Phe207fs)	~10th centile	Mild ID	Simple sentences; w = 16.5 m	4 m	GTC	Normal at seizure onset; slow background and occasionally atypical spikes	Slightly widen of subarachnoid space at 7 m	N.A.	
**5**	Female 17 m	c.256 C >T (p.Arg86Cys); c.13 G >T (p.Glu5Ter,211)	Normal	Moderate ID	Can not walk without help	5 m	GTC	Normal	Poor myelination at 5 m	**LEV** with **OXC**	
**6**	Male 14 m	c.409 T >G (p.Cys137Gly)	Normal	None	Normal	2 m	GTC	Epileptiform discharge in the right anterior temporal and middle temporal regions	Normal	**LEV** with **OXC**	
**7**	Male 1 y 8 m	c.264delC (p.Ileu88fs)	Normal	None	Normal	6 m	GTC	Normal	Normal	**LEV**	
**8**	Female 2 y	c.410 G> T (p.Cys137Phe)	<5th centile	None	Normal	6 m	GTC	Sharp waves in the frontal, central, middle temporal, and middle line regions	Normal	**VPA**	
**9**	Male 2 y	c.332 G >C (p.Arg111Pro)	Normal	Mild ID	Speaks only several words; cannot walk without help	5 m	GTC	Normal	Normal	**LEV**, **TPM** with **OXC**	
**10**	Female 1 y 3 m	c.332 G >C (p.Arg111Pro)	Normal	Mild ID	Nonverbal; cannot walk without help	5 m	GTC	Normal	Normal	**LEV**	
**11**	Female 6 m	c.368-2 A>G		Mild ID	Cannot sit	3.5 m	GTC	Normal	Normal	**TPM**	
**12**	Female 2 y	c.533_534insGT (p.Pro179fs)	<5th centile	Profound ID	ND	2 m	Facial clonic	Frequent right parietal spikes were present during sleep. Frequent semi-rhythmic generalized bifrontally predominant 2 c/s spike-and-wave complex were seen during wakefulness. Occasional generalized 10-Hz fast rhythms were seen during sleep	Cerebellar atrophy		(14)
**13**	Male 7 y	c.494A>G (p.His165Arg)	~5th centile	Profound ID	ND	3 d	Focal	Not acquired	Mega cisterna magna		
**14**	Male 17 y	c.139C>T (p.Arg47Ter)	~5th centile	Mild ID	Delayed speech; w = 18 m	11 m	Myotonic-atonic		Normal	LTG, OXC, TPM then **PDS**, **LTG** with **CLN**	(16)
**15**	Male	c.108dup (p.Thr37Tyrfs)	~5th centile	Mild ID	Speaks a few meaningful words; w = 17 m	Early infancy	Myoclonic and complex partial			**VPA**, **LEV**	(15)
**16**	Male 5 y	c.303C>A (p.Tyr101Ter)		Profound ID	Nonverbal; w = 5 y	6 m	^*^Myoclonic, atonic			**LTG, VPA,ZNS**	(17)
**17**	Male 6 y	c.303C>A (p.Tyr101Ter)		Profound ID	Nonverbal; w = 6 y	10 m	^*^Cyclonic, atonic			**LTG, VPA**	
**18**	Male 36 y	c.58G>T (p.Glu20Ter)		LD	Delayed speech; w = 17 m	1 y	GTC			**PHT** to 10 years of age	
**19**	Male 12 y	c.27del (p.Trp9Ter)		Profound ID	Single words; walked but stopped at 9 y	4 m	^*^Myoclonic; RSE			**VPA, CLN, RUF, ZNS, PHT**, PB, TPM, LCM, KD, **VNS**	
**20**	Male 9 y	c.73-2A>G			Spoke at 2 y; w = 23 m	26 m	Febrile then afebrile GTC; ESES		T2 hyperintensity and restricted diffusion of pontine central tegmental tracts		
**21**	Female 18 y	c.124C>T (p.Gln42Ter)			Fine motor delay that improved	4 m	Febrile, focal onset		T2 hyperintensity & restricted diffusion of pontine central tegmental tracts	**PB** then **OXC**	
**22**	Male 3.5 y	c.1A>G (p.Met1?)			Spoke at 28 m; w = 23 m	none					
**23**	Male 12 y	c.94G>A (p.Asp32Asn)		Moderate ID	Spoke at 2–3 y; 25 words at 5 y; w = 2–3 y	1.5 m	Febrile		Mild to moderate diffuse abnormal signal in the cerebral white matter,low volume ventral pons		
**24**	Male 26 y	c.542del (p.Asn181fs)		Severe ID	Nonverbal; now only with assistance; w = 7 y	10 m	^*^GTC myoclonic, atypical absences; RSE				
**25**	Female 17 y	c.101T>C (p.Phe34Ser)		Mild ID	Delayed speech; w = 15 m	none			Periventricular gliosis		
**26**	Male 21 m	c.78_83dup (p.Glu27_Asp28dup)		Mild ID	Spoke at 21 m; w = 15 m	5 m	^*^Absences, GTC		Two possible germinolytic cysts	**ZNS** or **VPA** with **LEV**	
**27**	Male 19 m	c.105T>A (p.Asn35Lys)			Does not babble; cannot sit unsupported	none					
**28**	Male 9 y	c.409T>C (p.Cys137Arg)			Spoke at 2 y, sentences at 5 y	6 m	Focal onset, GTC			**VPA** to 3 years of age	
**29**	Male 31 y	c.94G>A (p.Asp32Asn)		Moderate ID	Speaks only a few words; w = 2 y	2 y	^*^Absences, tonic-spasms				
**30**	Female 8 y	c.139C>T (p.Arg47Ter)		Mild ID	Simple sentences; fine motor delay; w = 15 m	2 y	^*^Drop attacks, head drops with staring of eyes		Poor coordination	**ETX**, **CLB**	
**31**	Female 11 y	c.558-2A>G		Mild ID	Spoke at 2 y;motor delays		^*^Focal onset, GTC, drop attacks			**LEV**, **LTG**	
**32**	Male 6 y	c.229G>A (p.Glu77Lys)		LD	Spoke at 21 m; w = 21 m	none					
**33**	Male 12 y	c.291G>A (p.Met97Ile)		Mild ID	Spoke at 3 y; w = 17 m	4 m	Focal onset, GTC				
**34**	Female 15 y	c.394_404del (p.M132fs)		Mild ID	Spoke at 2.5 y; w = 18 m	3 y	GTC				
**35**	Female 13 y	c.2T>A (p.Met1?)		Mild ID	Spoke at 3.5 y; w = 19 m	1 y	^*^			**KD**, **LEV** to 5 years of age	
**36**	Male 11 y	c.181G>T (p.Glu61Ter)		Mild ID	Delayed speech; delayed walking	2 m	^*^GTC			**PHT**, **LTG**, **FBM**	
**37**	Female 3.5 y	c.256C>T (p.Arg86Cys)		None	Spoke at 8–9 m; w = 13 m	14 m	Myoclonic; absences			**ZNS**	
**38**	Male 4.5 y	c.316T>G (p.Phe106Val)		Mild ID	Spoke at 2 y; 30 words at 4.5 y; w = 17 m	3–4 y	Febrile				
**39**	Male 22 y	c.557+1G>A		Severe ID	Spoke at 2.5 y; w = 18 m	2.5 m	^*^GTC, myoclonic, absences, tonic; RSE				
**40**	Male 12 y	c.94G>A (p.Asp32Asn)		Moderate ID	Delayed speech; w = 2 y	7 y	^*^Absences			**VPA**, **ETX**	
**41**	Female 4 y 2 m	c.410G>T (p.Cys137Phe)	Normal	Mild ID	Unstable to sit alone, unable to climb, unable to move toys with both hands (7 months)	5 m	Tonic-clonic	Normal	Normal	**VPA**	(18)
**42**	Male 3 y	c.494A>G (p.His165Arg)	~10th centile	Profound ID	Unsteady upright head and cannot talk	5 d	^*^Myoclonic	More sharp waves in the central, apical, and midline areas	Normal	**PB**, **TPM** with **LEV**	
**43**	Male 3 y 2 m	c.499delC (p.Leu167fs)	<5th centile	Moderate ID	Unsteady when walking alone (17 months)	14 m	Febrile	Normal	Normal	N.A.	
**44**	Male 1 y 2 m	c.292-2A>T	<5th centile	Moderate ID	Delayed speech; Unsteady upright head (8 months)	4 m	Tonic-clonic;	Sharp and slow waves in the central, parietal, occipital, middle, and posterior temporal regions	Bilateral frontotemporal epidural space slightly widened and a small amount of subdural effusion	**DAP**, **PB** with **OXC**	
**45**	Female 5 m	c.3G>A (p.Met1?)	Normal	Profound ID	Unsteady upright head	3 m	Tonic-clonic;	More sharp waves in the left area	Abnormal signal shadow in the right temporal, occipital parietal lobe, hippocampus, and splenium of the corpus callosum	**PB** with **LEV**	
**46**	Male 7 m	c.558-3T>G	Normal	Profound ID	Cannot sit alone and turn over	3 m	Tonic-clonic	Sharp wave, spike wave, and slow wave in the frontal, central, and temporal area	Normal	**PB**, **LEV** with **TPM**	
**47**	Female 7 m	C.494A>G (p.His165Arg)		Profound ID	Inability to life the head	Newborn	Myoclonic spasms	Normal	Normal		(19)
**48**	Male 5 y	c.94G>T (Asp32Tyr)		Moderate ID	w = 28.8	9 m	Atonic	Disorganized base activity	Normal		
**49**	Female 16 m	c.286C> T (p.Gln96Ter)		Mild ID	Delayed speech	<16 m	GTC	Generalized spike-wave discharges	Normal	**VPA**	(20)
**50**	Male 20 m	c.108dup (p.Thr37Tyrfs)		Mild ID	Short sentences	20 m	Tonic-clonic	Multifocal spikes at onset and diffuse sharp waves at follow-up		**VPA**	
**51**	Male 7 d	c.494A>G (p.His165Arg)		Profound ID	Delayed motor development	7 d	Focal-clonic	Paroxysmal multifocal activity	hypoplasia of the cerebellar worm, delayed myelinization, and a megacisterna magna	**LEV**	
**52**	Male 15 y	c.27G>A (p.Trp9Ter)		Mild ID	Mild speech impairments	7 m	GTC	multifocal spikes and generalized sharp waves	Normal	**VPA**	
**53**	Female 7 y	c.368–2A>G		Mild ID	Normal	10 m	GTC	some diffuse slow waves during sleep and drowsiness	Normal	**VPA**	
**54**	Female 18 y	c.181_183del (p.Glu61del)		Profound ID	Short sentences; delayed motor development	8 m	Febrile		low-lying spinal cord with adipose transformation of the Filum Terminale, colloid cyst of the third ventricle, microcephaly, hypoplasia of the corpus callosum and pons, and enlargement of the cerebrospinal fluid spaces	**VPA**	
**55**	Female 16 m	c.332G>C (p.Arg111Pro)		Profound ID	Delayed speech and motor development	<16 m	GTC	focal and multifocal discharges with burst suppression	highlighted gyral simplification and delayed myelination		
**56**	Female 5 y	c.116T>G (p.Leu39Arg)		Mild ID	Delayed speech	5 y	^*^Absence	Typical spike–wave complexes (3 Hz)	Chiari type 1 malformation and syringomyelia	ETX then LEV and ZNS	
**57**	Female 5 y	c.384_394del (p.Met132Leufs)			Delayed speech; w = 17 m	9 m	GTC		Normal	**VPA**	
**58**	Male 7 m	c.1A>G (p.Met1?)	<5th centile	Profound ID	cannot sit	3 m	GTC	Diffuse slow spike, multiple spike-slow and sharp waves discharge	Normal	**LEV** with **TPM**	Our case
**59**	Male 1 y 9 m	c.100delT (p.Phe34fs^*^16)	<5th centile	None	Normal	5 m	GTC	Slow background and occasionally atypical spike	Normal	**VPA** with **LEV**	
**60**	Male 5 y 1 m	c.332G>C (p.Arg111Pro)	<5th centile	Moderate ID	Speaking only several words; w = 28 m	1 y 1 m	GTC	Diffuse high-amplitude pike wave	Slightly widen of lateral ventricles	**LEV**	
**61**	Male 9 y 1 m	c.332G>C (p.Arg111Pro)	<5th centile	LD	Normal	none	N.A.	N.A.	Normal	N.A.	
**62**	Male 8 y	c.158_159insA (p.Asp55fs)	~5th centile	Profound ID	Short sentences; w = 22 m	2 m	Focal	Epileptiform discharge in the left anterior temporal and middle temporal regions	Normal	**LEV**	

∇Inheritance unknown; ^*^multiple medications or medically refractory; †anti-seizure medications were available for only some patients, **bold** indicates if the medication was felt to be useful; y, years; m, months; d, days; ASM, anti-seizure medication; EEG, electroencephalogram; CLN, clonazepam; ESES, electrical status epilepticus during slow-wave sleep; ETX, ethosuximide; FBM, felbamate; FTT, failure to thrive; GERD, gastroesophageal reflux disease; PDS, prednisolone; GTC, generalized tonic clonic; ID, intellectual disability; LCM, lacosamide; LD, learning disability; LEV, levetiracetam; LTG, lamotrigine; MRI, magnetic resonance imaging; OXC, oxcarbazepine; PB, phenobarbital; PHT, phenytoin; RSE, refractory status epilepticus; RUF, rufinamide; TPM, topiramate; VNS, vagal nerve stimulator; VPA, valproic acid; w, walked; ZNS, zonisamide; N.A., not assessed or not available.

Patient 1 was a Chinese boy of non-consanguineous parents with a birth weight of 3.5 kg and a gestational age of 38^+5^ weeks. He had his first focal seizure at 3 months of age, and seizures occurred 6 to 15 times a day for the first 10 days. Each seizure lasted for approximately 30 s to 5 min. At the time of writing, he was 7 months old and presented with a severely short stature (height, 63 cm <2.5 SD). He has been hospitalized three times due to repeated seizures without fever. The electroencephalogram (EEG) results were abnormal at 3 months of age, showing a diffuse slow spike, multiple slow spikes, and sharp wave discharges. The results of brain magnetic resonance imaging (MRI), metabolic assays, and chromosomal karyotype analysis were all normal. The Gesell Developmental Assessment Scale for Children was used at 4 months of age, and the patient was diagnosed with severe global developmental delay. The seizures were controlled from 3 months of age until the present time with levetiracetam (LEV) and topiramate (TPM).

Patient 2 was a 1-year-and-9-month-old boy who had epilepsy without intellectual and developmental delays. The patient had his first seizure at 5 months of age, and the type of seizure was generalized tonic-clonic seizure (GTCS) with a frequency of nine times a day, each lasting for approximately 10 s to 1 min. EEG abnormalities (a slow background and occasionally, an atypical spike) were observed. The seizures were controlled with valproate (VPA) and LEV treatment. The patient did not have a developmental delay, and the brain MRI results were normal.

**Figure 1:**
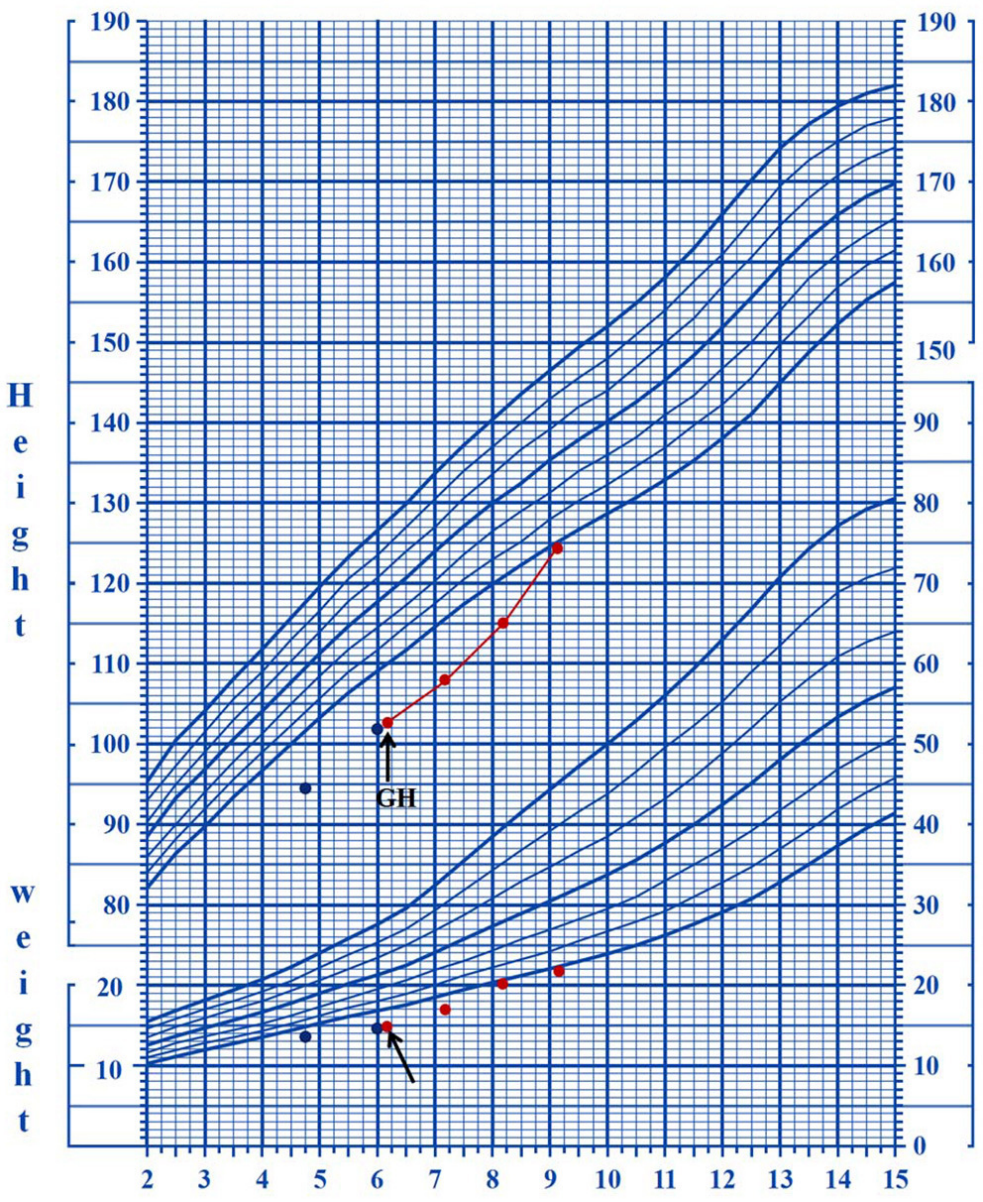
The growth curves of patient four who underwent rhGH treatment. The arrows indicate the starting date of the treatment.

*CSNK2B* mutations in patients 3 and 4 were identical; however, the patients had different clinical presentations. Patient 3 was a 5-year-and-1-month-old boy who presented with mild proportionate short stature with a Height Standard Deviation Score of −2.1 SD. GH deficiency was ruled out by the arginine clonidine GH stimulation test. The endocrine test results revealed an insulin-like growth factor 1 (IGF-1) level of 1,140 ng/ml (reference range, 49–283 ng/ml) and an insulin-like growth factor-binding protein 3 (IGFBP-3) level of 3.5 μg/ml (reference range, 1–4.7 μg/ml), and the highest GH level (insulin combined with arginine) in the stimulation test was 6.93 ng/ml. Patient 3 had epilepsy and mild DD. He started walking at 28 months and speaking at 3 years of age, and at the time of writing, he still could not construct long sentences. The results of the Wechsler Intelligence Scale for Children-IV test revealed that his full-scale IQ was 70. The patient had his first seizure at 1 year and 1 month of age, with a frequency of five times a day, each lasting for approximately 2 s. The seizures were characterized as myoclonic epilepsy. The patient was not taken to the hospital for treatment until half a year later, because of neglect by the parents. The EEG results were abnormal at 2 years of age with medium-to-high amplitude sharp waves in the frontal pole, as well as frontal and central regions. The brain MRI results revealed a slight widening of the lateral ventricles at 2 years of age, which was relieved at 4 years of age. Patient 4 was a 9-year-and-1-month-old boy without epilepsy and intellectual and developmental delays. At 6 years of age, he was taken to the hospital for genetic counseling of short stature (height, 102 cm, <3 SD) and learning disabilities. The results of the GH provocative test, which employed arginine and L-dopa as biomarkers, revealed that the highest GH level was 4.47 ng/ml (2.21 ng/ml at 0 min, 4.47 ng/ml at 30 min, 3.25 ng/ml at 60 min, 1.10 ng/ml at 90 min, 2.05 ng/ml at 120 min, 1.85 ng/ml at 150 min, and 0.88 ng/ml at 180 min), suggestive of a complete GH deficiency. The other hormonal measurements were an IGF-1 level of 115 ng/ml (reference range, 49–283 ng/ml) and an IGFBP-3 level of 2.8 μg/ml (reference range, 1–4.7 μg/ml). Recombinant human growth hormone (rhGH) therapy (0.2 mg kg−1 week−2, Subcutaneous injection) was initiated when the patient was 6 years and 1 month of age. The growth velocity was 5.31 cm/year before treatment (from 4 years and 11 months to 6 years of age). The growth velocity during the first year of treatment was 7.2 cm/year, and the height increased by 0.5 SD. By the end of the third year of treatment (9 years and 2 months of age), the height was 124.5 cm, and the height increased from −3.46 SD to −2 SD (Figure 1).

Patient 5 was an 8-year-old boy with epilepsy and moderate intellectual and developmental delays. He started walking at 22 months and talking at 2 years and 6 months of age. The Gesell Developmental Quotient Score was 52 at 5 years and 4 months of age. He continued to exhibit growth retardation, speech delays, comprehension deficits, and learning disabilities. The patient had his first focal seizure at 2 months and presented with tetanic twitching of the limbs with a frequency of 5 to 8 times a day, each lasting for approximately 30 s to 1 min. The results of brain MRI and EEG were normal. The seizures were controlled with LEV.

### Molecular Analysis

**Figure 2:**
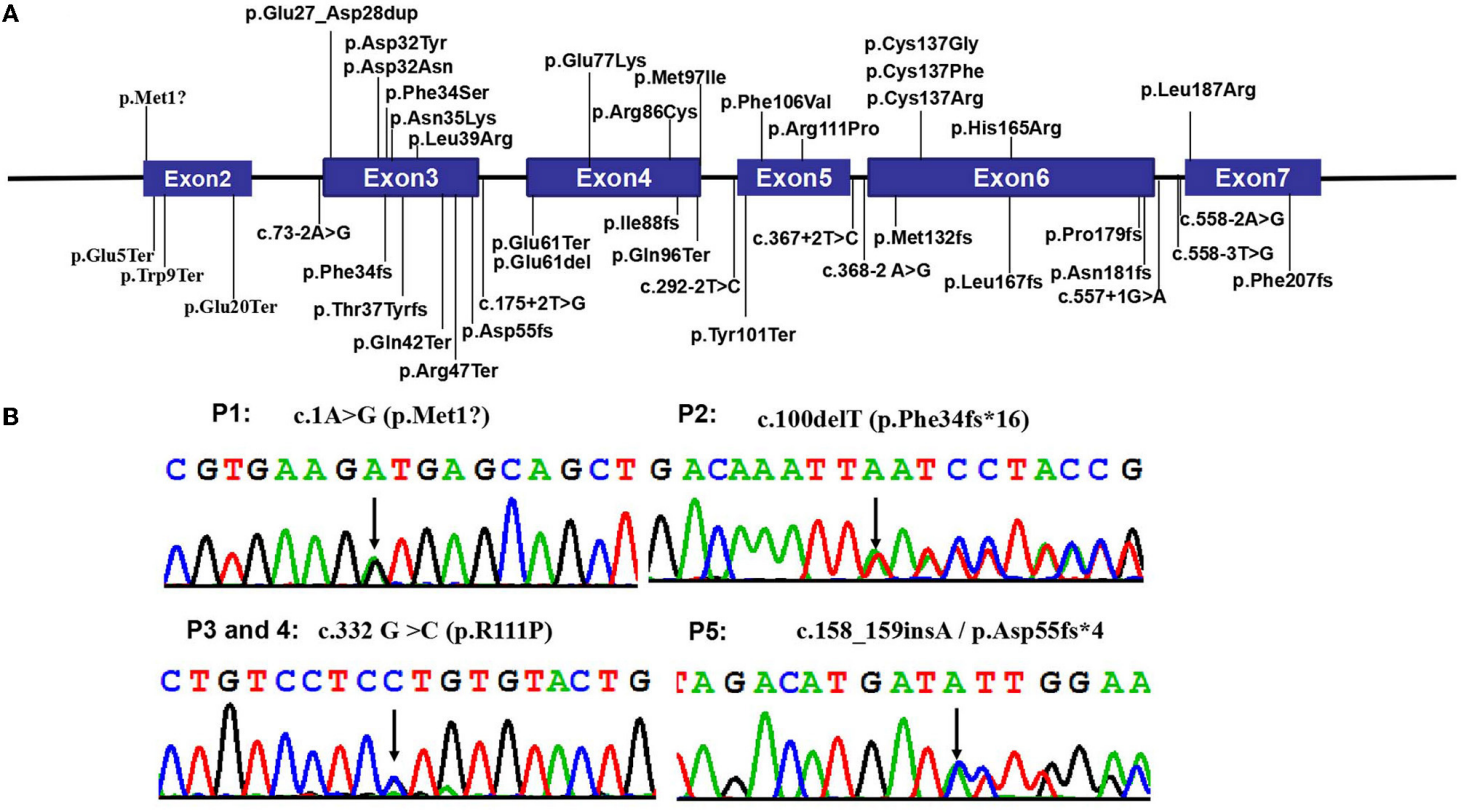
Pathogenic *CSNK2B* variants. **(A)** The distribution of all *CSNK2B* variants detected so far in the 57 reported patients and 5 in this study. Boxes represent six different exons as indicated, and solid lines connecting these boxes represent the introns of *CSNK2B* gene. The numbers above the boxes indicate the positions of the *CSNK2B* complementary DNA at the start-stop sites and exon-intron boundaries. Vertical lines represent the locations of missense (above the boxes) or deletion/nonsense/frameshift/splicing (below the boxes) variants. **(B)** The Sanger chromatograms of the detected variants in patients 1–5. Among them, P1 [NM_001320:c.1A>G (p.Met1?)] had a start loss variant, and P3 and P4 [c.332 G >C (p.R111P)] had missense variant; P2 [c.100delT (p.Phe34fs*16)] and P5 [c.158_159insA(p.Asp55fs*4)] had frameshift variants. Moreover, c.1A>G (p.Met1?) and c.332 G >C (p.R111P) were reported by Li et al. and Michelle et al.

By whole-exome sequencing, we detected heterozygous mutations in the *CSNK2B* gene in probands as follows: (RefSeq NM_001320.6): c.1A>G (p.Met1?) in patient 1, c.100delT (p.Phe34fs^*^16) in patient 2, c.332 G>C (p.R111P) in patients 3 and 4, and c.158_159insA (p.Asp55fs^*^4) in patient 5 (Figure 2). We confirmed the four mutations by Sanger sequencing and sequenced the parental samples to validate that the four variants were de novel. Specifically, c.100delT (p.Phe34fs^*^16) and c.158_159insA (p.Asp55fs^*^4) were novo variants, which were not deposited in the Human Gene Mutation database, 1000 Genomes Database, ClinVar database, and Single Nucleotide Polymorphism database. However, c.1A>G (p.Met1?) and c.332 G>C (p.R111P) were previously reported in affected individuals (2, 17). These *de novo* variants were predicted as deleterious through multiple functional prediction tools, including SIFT, PolyPhen 2.0, CADD, and MutationTaster (Table 2). All variants were classified as pathogenic according to ACMG/AMP standards and guidelines (Table 2).

**Table 2:** Predicted pathogenicity of *de novo* CSNK2B variants.

**Patient**	**Variant (NM_001320)**	**Inheritance**	**PolyPhen-2**	**SIFT**	**CADD**	**ACMG/AMP**
Patient 1	c.1A>G (p.Met1?)	DNM	PD (0.997)	D (0.049)	28.4	P(PVS1+PS2+PS4+PM2)
Patient 2	c.100delT (p.Phe34fs*16)	DNM	N.A.	N.A.	N.A.	P(PVS1+PS2+PM2)
Patient 3 and 4	c.332 G >C (p.R111P)	DNM	PD (1.000)	D (0.001)	28.6	P(PS2+PS4+PM2+PP3)
Patient 5	c.158_159insA (p.Asp55fs*4)	DNM	N.A.	N.A.	N.A.	P(PVS1+PS2+PM2)

*DNM, de novo mutation; D, deleterious or damaging; PD, probably damaging; N.A. not available; P, pathogenic*.

### Genotype–Phenotype Correlations

To date, a total of 57 patients with pathogenic CSNK2B mutations have been reported in the literature (1, 2, 14–20). Clinical and molecular features of the reported 62 patients and of our patients are summarized in Table 1. By extensive literature analysis, we compared the phenotypes of 24 patients with missense mutations and 38 patients with loss-of-function mutations (LoF; including deletions, duplications, frameshifts, nonsense mutations, start-loss mutations, and splice sites). The phenotypes were classified into five categories, namely, intellectual disabilities/learning disabilities, speech delay or disability, motor delay or disability, seizures, and short stature (Table 3). However, no significant differences in these phenotypes between the missense mutation group and the LoF mutation group were observed.

**Table 3:** Comparison of effects of variant type on clinical manifestations.

	**Missense mutations**	**LOF mutations^a^**	**All**
ID/LD	90.9%	94.1%	92.9%
Moderate to profound	50%	41.1%	44.6%
Speech delay or disability	84.2%	90.3%	88%
Motor delay or disability	73.9%	83.8%	80%
Seizures	83.3%	92.1%	88.7%
Age at seizure onset
<24 span month	80%	91.4%	87.3%
≥24 months	20%	8.6%	12.7%
Generalized tonic or tonic–clonic seizures	60%	68.6%	65.4%
Other types of seizures	65%	51.4%	56.3%
Medically refractory	38.5%	37.9%	38.1%
Short stature	60%	69.2%	65.2%

*ID, intellectual disability; LD, learning disability; LoF, loss of function*.

a *All deletions, duplication, frameshift, nonsense, start-loss, and splice site were considered predicted LoF for this table*.

## Discussion

POBINDS is a very rare autosomal dominant genetic disorder (1). Poirier et al. in 2017 provided the first report of mutations in the *CSNK2B* gene in two unrelated patients with neurodevelopmental abnormality and epilepsy; since then, a total of 57 patients with pathogenic mutations in this gene have been described (1, 2, 14–20). The most frequent manifestations of these subjects with mutations in the *CSNK2B* gene are epilepsy, developmental delays, and intellectual disabilities (Tables 1, 3). Patients are often diagnosed within the first 2 years of life, and the severity of the phenotype is highly variable (Table 1). In the present study, five individuals were diagnosed as POBINDS by performing whole-exome sequencing. These patients showed the common phenotypes associated with POBINDS, including varying degrees of ID/LD, developmental delay with or without seizures, and growth abnormalities.

Neurodevelopmental disabilities are a hallmark of CSNK2B gene deficiency. Many subjects (including our patients) suffer from delays that span multiple areas. For instance, most patients have delayed motor development, more than four-fifths of the patients have speech deficits, and one-thirds of patients have cognitive impairments that range from moderate to profound (1, 2, 14–20) (Table 3). Seizures, which start in infancy, are observed in approximately 88.7% of patients (Table 3). Different types of seizures have been reported, with GTCS as the most common type (Table 3). In this study, three out of four patients with epilepsy presented with GTCS. Although many patients have severe epilepsy with recurrent episodes of refractory status epilepticus (17), four patients in this study became seizure-free with LEV or combination therapy. Intellectual disabilities are usually associated with seizure severity. However, the intelligence and development of POBINDS patients with intellectual/developmental disabilities and treatable epilepsy do not always improve after the seizures are controlled with therapy, and some patients with normal intelligence still experience seizures, indicating that intellectual/developmental disabilities and seizures may be comorbidities. Further studies are needed to define the genotypic and phenotypic determinants, as well as the mechanisms of action.

In addition to intellectual/developmental disabilities and epilepsy, short stature may be another common feature of the disease. Short stature has been observed in most patients (15/23, 65.2%), including our patients; however, it was not reported by Ernst et al. (17). Human GH is often used to treat growth disorders in children with GH deficiency. Thus, far, only one patient with CSNK2B deficiency has been tested and ruled out for GH deficiency (16). The patient received GH therapy, and his height increased significantly. Of the five patients in this study, two subjects (patients 3 and 4) were subjected to the GH stimulation test, and the results revealed that patient 3 showed partial GH deficiency and patient 4 showed complete GH deficiency. Patient 4 underwent rhGH treatment for 3 years and showed a good response with no side effect. This study provides further evidence for GH therapy in patients with mutations in the *CSNK2B* gene.

To date, 46 *CSNK2B* gene mutations have been identified in 62 Poirier–Bienvenu neurodevelopmental syndrome patients, including 8 splice site mutations, 9 frameshift mutations, 15 missense mutations, 8 nonsense mutations, 1 in-frame duplication, 1 in-frame deletion, and 3 start-loss mutations. CSNK2B deficiency is associated with clinically heterogeneous deficits, including intellectual disabilities, multiple congenital anomalies, development delays, seizures, and short stature (1, 2, 14–20) (Table 1). We tried to correlate the mutations in the CSNK2B gene with the clinical symptoms of Poirier–Bienvenu neurodevelopmental syndrome patients. We analyzed the genetic and clinical information of all reported patients and our cases (Tables 1, 3). Mild or severe phenotypes can be evident in patients with missense or LoF mutations, and although severe phenotypes were associated more often with LoF mutations than with missense mutations, this finding was not statistically significant. Even patients with the same mutation can have different phenotypes. For instance, patients (including our patients) with the L111P mutation have varying degrees of intellectual impairment with delayed cognitive, language, and motor development, and growth abnormalities with or without seizures (Table 1). As such, the clinical features of patients with heterozygous mutations in the CSNK2B gene are highly variable with no obvious genotype–phenotype correlation. It is unclear how these mutations cause neurodevelopmental deficits and epilepsy. Nakashima et al. reported that the disease can be explained by several mechanisms, although LoF mutations are responsible for CSNK2B haploinsufficiency (15). Furthermore, the reduced expression of CSNK2B results in fewer CK2 tetrameric complexes. On the other hand, missense mutations and other types of mutations, such as p.His165Arg and p.Pro179Tyrfs^*^49, can have a dominant negative effect and impair CK2 enzyme activity. Further functional studies are needed to enhance our understanding of the disease and its mechanisms of action.

## Conclusion

In summary, we presented the clinical phenotypes of five unrelated Chinese patients with *de novo* heterozygous mutations in the *CSNK2B* gene. This study expands the mutation spectrum of Poirier–Bienvenu neurodevelopmental syndrome. This is the first report of c.158_159insA/p.Asp55fs^*^4 and c.100delT (p.Phe34fs^*^16) mutations in the *CSNK2B* gene. Our study also summarizes the phenotypes of patients with *CSNK2B* deficiency and reveals no significant correlation between genotype and phenotype, thereby providing a foundation for clinical diagnosis in the future. Long-term recombinant GH treatment appears safe and effective; however, additional cases should be examined to fully evaluate the benefits of GH therapy.

## Data Availability Statement

The datasets presented in this study can be found in online repositories. The names of the repository/repositories and accession number(s) can be found at: https://www.ncbi.nlm.nih.gov/, PRJNA778391.

## Ethics Statement

The studies involving human participants were reviewed and approved by Department of Genetic Metabolic Central Laboratory of Guangxi Zhuang Autonomous Region, Women and Children Care Hospital. Written informed consent to participate in this study was provided by the participants' legal guardian/next of kin. Written informed consent was obtained from the individual(s), and minor(s)' legal guardian/next of kin, for the publication of any potentially identifiable images or data included in this article.

## Author Contributions

QY and SH designed the study. QY and JL gathered clinical information from the family members and drafted the manuscript. ZQ, SY, QZ, FS, and SO performed the sequencing, as well as analyzed, and interpreted the data. All authors coordinated the study coordination and revised the manuscript, read, and approved the final version of the manuscript.

## Funding

This research was supported by the Health Department of Guangxi Province (Grant Nos. ZJC202001, Z20210440, and Z2015238), Guangxi Medical High-level Backbone Talents 139 Plan (G202003023), and Guangxi Key Research and Development Program (GuikeAB17195004).

## Conflict of Interest

The authors declare that the research was conducted in the absence of any commercial or financial relationships that could be construed as a potential conflict of interest.

## Publisher's Note

All claims expressed in this article are solely those of the authors and do not necessarily represent those of their affiliated organizations, or those of the publisher, the editors and the reviewers. Any product that may be evaluated in this article, or claim that may be made by its manufacturer, is not guaranteed or endorsed by the publisher.
